# Research on flexibility-enhanced planning for renewable energy systems with supply-demand uncertainties

**DOI:** 10.1371/journal.pone.0331284

**Published:** 2025-09-30

**Authors:** Tianhe Sun, Xilai Bai, Xiaoyi Qian, Baoshi Wang

**Affiliations:** Shenyang Institute of Engineering, Shenyang, Liaoning, China; Aalto UniversityssFINLAND

## Abstract

Due to the rapid development of intermittent renewables and emergence of new types of load, flexibility becomes a crucial element for reliable and cost-effective power system operation. This paper proposes a dual-index flexibility evaluation metric (incorporating flexibility margin and insufficiency probability) that considers the dynamic supply-demand balance under net load uncertainty. Additionally, a robust two-stage power planning model is presented to enhance system flexibility, utilizing the proposed metric. The model is solved iteratively using the column generation algorithm and strong duality theory. Case studies on a Northeast China power grid demonstrate that, by optimally configuring generation and storage capacity guided by flexibility and other indicators, the proposed method reduces curtailment/load shedding costs and system flexibility insufficiency probability by 45% and 4.3% respectively. Furthermore, incorporating energy storage planning achieves additional significant reductions of 27% and 1.1% in these metrics, verifying its effectiveness. Comparative analysis confirms the superiority of the proposed robust-probabilistic hybrid model over traditional uncertainty quantification methods in balancing computational efficiency, risk control, and curtailment reduction.

## 1. Introduction

Generation portfolios are changing significantly in many power systems world-wide. The growing penetration of renewable energy, such as wind and solar power, poses a significant challenge of meeting the increased flexibility demand due to the increasingly uncertain and variable nature of power supply and demand [1]. In the con-text of these high-penetration renewable energy systems, flexibility is widely recognized as a crucial performance metric, affecting system safety, reliability, and economic feasibility [2].

Ensuring sufficient flexibility is one of the key issues during the planning stage. The concept of power system flexibility has been proposed as early as the 1990s [3]. With the evolution of power systems, the uncertainties faced by the power systems have gradually increased, and the definition of flexibility has also been constantly ex-tended with the development of power systems. Power system flexibility refers to the ability to maintain safe and reliable operation of the system, while considering the economic operation of the system, by quickly responding to supply-side random out-put fluctuations and demand-side random output fluctuations represented by demand response, while meeting the output and ramping restrictions of the system units [4]. Power system flexibility comes from the available upward/downward adjustment capability of various flexibility resources, including flexible generators, inter-regional transmission, demand response, energy storage, and even renewable energy sources themselves [5–9]. The common factors to consider in assessing flexibility include ramp rate, power, and unit capacity [10,11]. A unified framework for measuring the flexibility that various types of flexibility resources can provide in power systems has been proposed in [12].

Furthermore, to accurately assess the flexibility capabilities of a system, it is necessary to take into account the uncertain factors present in the system, such as the output of renewable energy, random fluctuations in load demand, and changes in operating conditions resulting from industrial production. The uncertainty in wind and photovoltaic power output as well as in load demand is a crucial concern in system planning. Currently, uncertainty is mainly addressed using methods such as interval analysis, robust optimization, and scenario analysis. Uncertainty quantification primarily employs methods such as Conditional Value at Risk and Information Gap Decision Theory. For long-term uncertainty, the scenario analysis method is primarily used to simulate typical scenarios [13,14]. For short-term prediction errors, interval analysis and robust optimization methods are commonly used [15,16]. Due to the fact that it is not necessary to establish accurate models for wind power, photovoltaic power generation, and load forecasting, but only to capture their ranges of fluctuations, the application of robust optimization in the planning model is more convenient. The key issue is how to ensure the economic and flexibility optimality of the planning and operation scheme while ensuring safety and reliability.

Consideration of flexibility in system planning is a complex optimization problem that involves different components and stages. Before planning, it is necessary to estimate the current level of flexibility and determine the current and future flexibility requirements. When there is a lack of flexibility in the system, investment in flexibility resources needs to be increased. The urgent demand for flexibility may change the preference for flexibility resource substitution options, considering cost-effectiveness and incubation period. Alternatively, the current flexibility needs may be satisfied first, followed by future flexibility planning. Currently, research of flexibility planning concerning power sources primarily focuses on flexibility characterization [17]. In [18], power system flexibility is measured using the same method as Loss of Load Expectation (LOLE) to determine whether the system’s flexibility resource capacity is sufficient, while taking into account operational constraints. In [19], CVaR is adopted to control the tail risk of loss distributions for managing extreme events, thereby conducting risk-averse planning of multi-energy microgrids. In [20], IGDT is utilized to handle extreme uncertainties with missing probabilistic information, further addressing recovery issues under typhoon disasters. However, CVaR requires accurate probability distributions and involves high computational complexity. When solving flexibility planning problems in high-penetration renewable energy systems, these methods struggle to balance computational efficiency and risk control simultaneously. In [21–23], a probabilistic assessment indicator, named insufficient ramp rate expectation (IRRE), is proposed to characterize the needs of flexibility for very operation point. The above-mentioned system planning approaches that consider flexibility are effective in scenarios with low to moderate renewable energy penetration, as they mainly improve the classical power supply planning method based on the net load envelope and focus on planning for a certain type of flexibility resource.

To summarize, this paper examines the balance between the flexibility of supply and demand in high-penetration renewable energy systems, taking into account uncertainties related to wind, solar, and load. It is proposed to quantify system flexibility through dual indicators of flexibility margin and insufficiency probability. and uses robust optimization theory to develop a two-stage planning model that considers both flexibility planning and economic efficiency. The model is further applied to enable an optimal planning procedure that aims to achieve optimal system economy and flexibility. A case study is conducted on a power grid in northeastern China to demonstrate the effectiveness of the proposed solution.

## 2. Flexibility supply-demand balance

In energy systems with high levels of variable renewable energy sources, maintaining a dynamic balance between flexibility supply and demand is crucial. If the flexibility supply falls short of meeting the demand in the upcoming period, the power system may need to increase power output or decrease load demand on the demand side, which are known as supply-up flexibility and demand-down flexibility, respectively. Conversely, if the flexibility supply exceeds the demand, the power system may need to reduce power output, store excess energy, or increase load demand on the demand side, which are referred to as supply-down flexibility and demand-up flexibility. Inadequate supply-down flexibility and demand-up flexibility may lead to the waste of valuable resources such as wind and solar. Table 1 outlines the principles of flexibility demand in the same direction.

**Table 1:** Principles of flexibility demand in different directions.

Supply-Demand Relationship	Flexibility Demand	Adjusting Measures	Example
Flexibility supply is less than demand	Supply flexibility upward	Increase output of flexible resources	Increase output of thermal **power** plants, discharge energy storage
	Demand flexibility downward	Reduce demand on the load side	Interrupt or shift load demand in demand response
Flexibility supply is greater than demand	Supply flexibility downward	Reduce output of flexible resources	Deeply peak adjust thermal power
	Demand flexibility upward	Increase load demand	charge energy storage

### 2.1. Analysis of flexibility supply

#### 2.1.1. Conventional generation units.

Conventional generation units generally refer to hydro and thermal power units. These units can adjust their output power within a certain range between the maxi-mum and minimum technical output at a certain rate. Conventional units can provide flexibility in proportion to their installed capacity, and the flexibility resources they provide can be coupled with the supplied electricity.


$$f_{g}^{+}(t,\tau )=\min(R_{g}^{+}\tau ,P_{g}^{\max}-P_{g}(t))$$ (1)

$$f_{g}^{s-}(t,\tau )=\min(R_{g}^{-}\tau ,P_{g}(t)-P_{g}^{\min})$$ (2)

Where τ represents the time scale, once a time scale is given, the flexibility of a unit is only related to its output status, $R_{g}^{+},R_{g}^{-}$ respectively represent the ramping-up rate and ramping-down rate of conventional power plants, and $P_{g}^{max},P_{g}^{min}$ represent the maximum and minimum technical output of the conventional power plants. $P_{g}(t)$ represents the output power of conventional power plants at time t.

#### 2.1.2. Energy storage system.

Energy storage systems (ESS) have the ability to both absorb and supply electricity. Therefore, when charging the ESS, they are considered as providing downward flexibility resources, whereas when discharging the ESS, they are considered as providing upward flexibility resources. The up and down flexibility of ESS is influenced by fac-tors such as its capacity and charging/discharging speed. The formulas for upward and downward flexibility of ESS are as follows:


$$f_{S}^{s-}(t,\tau )=min(P_{c}(t),\frac{\delta_{SOCmax}-\delta_{SOC}(t)}{\tau })$$ (3)

$$f_{S}^{s+}(t,\tau )=min(P_{d}(t),\frac{\delta_{SOC}(t)-\delta_{SOCmin}}{\tau })$$ (4)

Where $f_{S}^{s+}(t,\tau )/f_{S}^{s-}(t,\tau )$ represent the upward and downward flexibility provided by the ESS, respectively. $P_{d}(t)/P_{c}(t)$ are the discharging and charging power of the ESS, respectively. $\delta_{SOC}(t)$ is the remaining energy in the ESS at time t. $\delta_{SOCmin}/\delta_{SOCmax}$ are the lower and upper limits of the state of charge constraints of the ESS, respectively.

#### 2.1.3. Curtailment and load shedding.

The demand of flexibility in a power system mainly comes from the load and renewable energy. In extreme operating conditions, the power system has to meet flexibility demand through curtailment or load shedding. Curtailment of renewable energy sources belongs to the down-regulation flexibility resources, while load shedding provides up-regulation flexibility resources. These two methods are means of ensuring system security when there is a flexibility deficit, rather than an active regulatory resource. Its weight is determined by the system tolerance:

Curtailment reflects the priority of renewable energy accommodation, and its weight is positively correlated with the penalty cost; load shedding represents the bottom line of power supply reliability, and its weight must meet reliability standards such as EENS (for example, the national standard requires it to be < 0.01%).


$$f_{RE}^{s+}(t,\tau )=\sum_{i\in \Omega }P_{RE-i}(t)$$ (5)

$$f_{F}^{s-}(t,\tau )=P_{L-cut}(t)$$ (6)

The variables in the above equation represent the following: $P_{RE-i}(t)$ denotes the power curtailment or reduction of the i-th renewable energy unit, Ω represents the set of renewable energy units that can be curtailed or the curtailed power, and $P_{L-cut}(t)$ represents the power reduction of the load.

### 2.2. Analysis concerning flexibility demand

Flexibility demand is mainly caused by fluctuations in net load and forecasting errors. It is assumed that the net load fluctuation process is a linear change over a certain time scale. In this paper, the power in each time period within a certain time scale is divided into the sum of the predicted value and the error value and considered as a random variable that follows a predetermined probability distribution. The general formula is shown in Equation (7):


$$P=P^{\prime }+\Delta P$$ (7)

Assuming a power forecast value of with an error that follows a certain probability distribution F(ΔP), a fluctuation range $\left[\Delta P^{-},\Delta P^{+}\right]$ can be obtained. This paper uses a confidence interval estimation method based on the power error distribution to extract the power interval variable, ensuring that the solution meets a certain confidence level. By knowing the probability distribution of the power error, a segment can be taken within the error fluctuation range, which results in a specific confidence level.

In this paper, the prediction error is treated as a random variable that follows a normal distribution with a mean of 0 and a standard deviation proportional to the predicted value at a certain time scale. Therefore, the probability density function of the output prediction error is given as follows:


$$f(\Delta P_{r})=\frac{1}{\sqrt{2\pi }\sigma }e^{-\frac{{\left(\Delta P_{r}\right)}^{2}}{2\sigma^{2}}}$$ (8)

σ(t)=δP(t) (9)

In the equation, the variance of wind power output prediction error is generally taken as the ratio of the standard deviation to wind power predicted output α%, i.e., ${\sigma_{W}}^{2}=P_{W}^{\prime }\times \alpha \%$,; the variance of PV power output prediction error is generally taken as the ratio of the standard deviation to PV predicted output β%, i.e., ${\sigma_{PV}}^{2}=P_{PV}^{\prime }\times \beta \%$; the variance of load prediction error is generally taken as the ratio of the standard deviation to load predicted output γ%, i.e., ${\sigma_{L}}^{2}=P_{L}^{\prime }\times \gamma \%$, where is a coefficient.

The flexibility demand of the power system can be expressed as the system’s ability to adjust and cope with uncertain changes in net load, which depends on the time series fluctuations of both the load *Lt* and renewable energy $P_{r,t}$, as shown in Equation (10).


$$P_{NL,t}=L_{t}-P_{r,t}$$ (10)

$$f^{n,+/-}=P_{NL,t+\Delta t}-P_{NL,t}$$ (11)

Where $P_{NL,t}$ denote the net load power at each time step in the system.

### 2.3. Flexibility indicators

Flexibility indicators are a key consideration in power system planning as they represent the overall capability of flexibility supply resources to meet flexibility demands. In high-penetration renewable energy systems, flexibility indicators should reflect the system’s ability to respond to changes in renewable energy or load uncertainty. The insufficient flexibility probability and the selection of flexibility margin are defined as flexibility evaluation indicators.

#### 2.3.1. Flexibility margin.

Traditional studies typically calculate flexibility margins using a fixed confidence interval, ignoring the time-series characteristics of prediction errors. This paper establishes a dynamic mapping relationship between the statistical characteristics of prediction errors and confidence intervals:


$$\Delta \alpha_{r}=\Phi^{-1}(1-\frac{\sigma_{r}}{\mu_{r}})$$ (12)

In the equation, $\Phi^{-1}$ denotes the inverse function of the standard normal distribution, and $\sigma_{r}/\mu_{r}$ represents the coefficient of variation of renewable energy prediction errors. This formula converts prediction accuracy $\left(\sigma_{r}\propto P_{forecast}\right)$ into a real-time confidence level $\alpha_{r}$.

The flexibility margin refers to the range of flexibility that a system can provide under certain installation conditions, and its calculation method can be expressed as:


$$\Delta F\;=\;\sum_{k\in K}R_{k}S_{k}\times \alpha_{r}\;\cdot \beta_{l}$$ (13)

In the equation, $\sum R_{k}S_{k}$ represents the integrated ramp capability of resources such as thermal power and energy storage (where $R_{k}$: ramp rate;$S_{k}$: available capacity ratio). $\alpha_{r}\;$ and $\beta_{l}\;$ are the corresponding confidence levels.

#### 2.3.2. Probability of inadequate flexibility.

Although the required level of system flexibility may vary depending on the specific circumstances, the flexibility constraints can be defined in a uniform manner. In planning models, the flexibility supply and demand can be defined as single random variables, and the flexibility provided by various resources can be integrated into the system’s overall set of random variables. For high-renewable power systems, the flexibility supply and demand can be described using cumulative probability. The probability of inadequate flexibility is defined as the probability that the system’s flexibility supply is less than the flexibility demand.


$$p_{S-insuf}=\mathrm{Pr}\left\{f^{s,+/-}(t,\tau )<f^{n,+/-}(t,\tau )\right\}$$ (14)

In the equation, $p_{S-insuf}$ represents the probability of insufficient system flexibility. In the planning model, flexibility supply and flexibility demand are respectively defined as a single random variable, and the flexibility provided by various resources can be integrated into all random variables of the system. Then, the system flexibility margin can be expressed in the form of probability.

The calculation of the system’s probability of insufficient flexibility, denoted as $P(F_{sup}<F_{dem})$, requires simultaneous consideration of the distribution of regulation capabilities of multiple flexibility resources (such as thermal power, energy storage, and wind/solar curtailment) as well as the stochastic characteristics of flexibility demand. This paper proposes a resource integration framework based on joint probability distribution:


$$P(F_{sup}<F_{dem})=\int \int_{0}^{\infty }f_{F_{dem}}(x)\cdot [\prod {\mathrm{\backslash nolimits}}_{k=1}^{K}F_{F_{sup,k}}(x)]d_{x}$$ (15)

In the equation, K represents the number of flexibility resource types, and $F_{F_{sup,k}}(x)$ denotes the cumulative distribution function of the regulation capability of the *k*-th type of resource.

**Fig 1:**
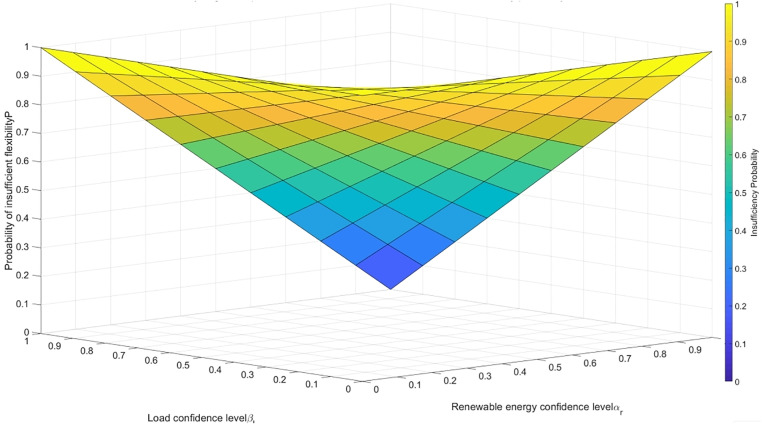
Synergistic impact of dual-index confidence intervals on insufficiency probability.

To clarify the synergistic relationship between the margin index ΔF and the insufficiency probability P, Fig 1 illustrates the nonlinear impact of confidence interval parameters $\left(\alpha_{r},\beta_{l}\right)$ on $P(F_{sup}<F_{dem})$:

The dual-dimensional flexibility indices proposed in this paper (margin ΔF and insufficiency probability P) are dynamically linked through confidence interval parameters $\alpha_{r}$ and $\beta_{l}$. As can be observed from Fig 1, the insufficiency probability P exhibits a nonlinear relationship with $\alpha_{r}$ and $\beta_{l}$: when $\alpha_{r}$ or $\beta_{l}$ is low, P rises rapidly, indicating that the deterioration of a single confidence interval will significantly increase system risks. Therefore, in the planning process, it is necessary to coordinate and optimize the two parameters rather than fix their values as in traditional methods.

## 3. High-penetration renewable energy system power flexibility enhancement planning model

### 3.1. Overall planning approach

A coordinated bi-level optimization model is proposed in this paper to enhance power flexibility in high-penetration renewable energy systems. The model is multi-objective and consists of two layers. The upper layer is the planning and configuration layer that aims to optimize economic results based on factors such as annual electricity demand, renewable energy penetration rate, and balance of power flexibility supply and demand. Flexible resources such as wind, solar, hydro, and energy storage are planned and constructed with fixed capacities in this layer to determine the optimal planning and construction scheme for production operation simulation at the annual scale. The lower layer is the production operation simulation layer, which optimizes the system’s operation economy under the determined system structure in the upper layer. Multiple production scenarios are simulated to calculate the optimal eco-nomic operation strategy, and the system operating parameters for each scenario are returned to the upper layer for evaluation and optimization of the planning scheme. Through this iterative process, the optimal construction plan for flexible resources can be obtained.

### 3.2. Planning layer model

#### 3.2.1. Objective function.

The objective function of the high-penetration renewable energy system resource investment decision model is to minimize the investment and annual operation costs of newly installed resources while satisfying the constraints of power balance, renewable energy penetration rate, carbon emission, and flexibility margin. The decision variables include the capacity of new power and flexibility resources.


$$f_{1}=\min C_{total}=\min(C_{inv}+C_{op})=\min(C_{inv}+\sum_{s\in \Gamma }\phi_{s}C_{op,s})$$ (16)

The objective function for the planning layer model is defined as $f_{1}$, where $C_{inv}$ is the annual investment and construction cost and $C_{op}$ is the annual operating cost.Γ rep-resents the set of operating scenarios, $\phi_{s}$ is the probability of scenario s occurring, and $C{\mathrm{\backslash nolimits}}_{op,s}$ is the annual operating cost under scenario s.


$$C_{inv}=(\sum_{t=1}^{T}\sum_{i\in \Omega {\mathrm{\backslash nolimits}}_{s}^{new}}x_{it}c_{it}P{\mathrm{\backslash nolimits}}_{it}^{new}+\sum_{t=1}^{T}\sum_{i\in \Omega {\mathrm{\backslash nolimits}}_{F}}x_{jt}c_{jt}P{\mathrm{\backslash nolimits}}_{jt}^{new})CRF$$ (17)

$$CRF=\frac{\sigma (1+\sigma ){\mathrm{\backslash nolimits}}^{N{\mathrm{\backslash nolimits}}^{i,j}}}{\left(1+\sigma \right){\mathrm{\backslash nolimits}}^{N{\mathrm{\backslash nolimits}}^{i,j}}-1}$$ (18)

Where T is the planning horizon; $x_{it}$ and $x_{jt}$ are binary variables indicating whether power unit i and flexibility resource j are newly built; $\Omega {\mathrm{\backslash nolimits}}_{S}^{new}$ is the set of existing power sources, including conventional thermal power, wind power, and photovoltaic power, and $\Omega {\mathrm{\backslash nolimits}}_{F}$ is the set of newly built flexibility resources; $c_{it}/P{\mathrm{\backslash nolimits}}_{it}^{new}$ and $c_{jt}/P{\mathrm{\backslash nolimits}}_{jt}^{new}$ are the investment cost and unit investment cost of power source i and flexibility resource j in year t, respectively; CRF is the investment cost recovery factor for new units, $N{\mathrm{\backslash nolimits}}^{i,j}$ and are the service life of various types of power sources and flexibility resources; σ is the discount rate.

#### 3.2.2. Constraints.

1) Power balance constraints.

$$\sum_{i=1}^{m}\lambda_{it}P_{it}+\sum_{j=1}^{n}\lambda_{jt}P_{jt}\geq L{\mathrm{\backslash nolimits}}_{t,\max}(1+\xi {\mathrm{\backslash nolimits}}_{t})$$ (19)

Where m and n represent the types of power and flexibility resources, $P{\mathrm{\backslash nolimits}}_{it}^{new}$ and $P{\mathrm{\backslash nolimits}}_{jt}^{new}$ represent the final installed capacity of the system’s power and flexibility resources. $\lambda_{t}$ represents the confidence level of the output of each type of power and flexibility resource in the t -th year; $\xi {\mathrm{\backslash nolimits}}_{t}$ represents the load reserve capacity coefficient of the system, and $L{\mathrm{\backslash nolimits}}_{t,\max}$ represents the maximum demand for the t -th year.

2) Electricity balance constraint

$$\left(\sum_{i\in \Omega {\mathrm{\backslash nolimits}}_{s}}E_{it}+\sum_{i\in \Omega {\mathrm{\backslash nolimits}}_{F}}E_{jt})(1-\varepsilon_{loss})\geq E_{t}(1+R_{et}\right)$$ (20)

Where $E_{it}$ and $E_{jt}$ represent the current power generation of power and flexibility resources, $\varepsilon_{loss}$ represents the power loss rate, which is generally set to 10%, and $E_{t}$ represents the predicted power demand in the t -th year, and $R_{et}$ represents the reserve rate of various power sources.

3) Renewable energy penetration rate constraint

$$\partial_{rt,\min}E_{it}\leq \sum_{t=1}^{T}\sum_{r\in \Omega R}E_{rt}(1-\varepsilon {\mathrm{\backslash nolimits}}_{loss})\leq \partial_{rt,\max}E_{it}$$ (21)

Where $\partial_{rt,\min},\partial_{rt,\max}$ represent the minimum and maximum requirements for renewable energy penetration rate during the planning period, ΩR represents the energy power source set, and $E_{rt}$ represents the power generation of renewable energy r in the t -th year.

4) Flexibility margin constraint

$$F_{req}=S_{r}\cdot \alpha_{r}+S_{l}\cdot \beta_{l}$$ (22)

$$\Delta F\geq F_{req}$$ (23)

Where $S_{r}\;$ and $S_{l}\;$ are the fluctuation reference values of renewable energy and load (in MW), $\alpha_{r}\;$ and $\beta_{l}\;$ are the corresponding confidence levels.

### 3.3. Operation layer model

#### 3.3.1. Objective function.

The operation layer model optimizes and solves the sub-problems for typical operation scenarios based on the system unit investment portfolio determined by the planning layer model. The objective of the operation layer model is to minimize the annual operating cost for economic optimal production simulation. The objective function of the operation layer model is given by:


$$f_{2}=\min\left\{C_{oper,s}\right\}=\min\left\{C_{op,s}+C_{f,s}+C_{curt,s}\right\}$$ (24)

In the equation, $C_{op,s}$, $C_{f,s}$, $C_{curt,s}$, respectively represent the system production and operation cost, fuel and environmental cost, renewable energy curtailment penalty cost.

Production and operation and maintenance cost, $C_{op,s}$, is shown in Equation (23):


$$C_{op}=\sum_{t=1}^{T}\sum_{i\in \Omega {\mathrm{\backslash nolimits}}_{s}}o_{it}P{\mathrm{\backslash nolimits}}_{it}+\sum_{t=1}^{T}\sum_{j\in \Omega {\mathrm{\backslash nolimits}}_{F}}o_{jt}P{\mathrm{\backslash nolimits}}_{jt}$$ (25)

In the equation, $O_{it}$ and $O_{jt}$ are the fixed operation and maintenance costs of all power sources and flexible resource units in the t-th year, respectively.

Fuel and environmental cost, $C_{f}$, is shown in Equation (24):


$$C_{f}=\sum_{t=1}^{T}\sum_{i\in \Omega {\mathrm{\backslash nolimits}}_{s}}(c_{fuel}E_{gt}+e_{co2}c_{co2}E_{gt})$$ (26)

In the equation,$E_{gt}$ is the power generation of conventional thermal power units, $C_{fuel}$ is the fuel cost, $E_{co2}$ is the carbon emission intensity, $C_{co2}$ and is the carbon emission cost.

Renewable energy curtailment cost, $C_{f}$, is shown in Equation (25):


$$C_{curt}=\sum_{t=1}^{T}\sum_{r\in \Omega R}\omega_{curt}^{r}E_{rt}^{curt}$$ (27)

In the equation, $\omega_{curt}^{r}$ is the unit penalty cost for renewable energy curtailment, and is the amount of renewable energy curtailment.

#### 3.3.2. Constraints.

1) Output limits constraints

$$\delta {\mathrm{\backslash nolimits}}_{i}^{\min}P_{it}\leq P_{it,d}\leq \delta {\mathrm{\backslash nolimits}}_{i}^{\max}P_{it}$$ (28)

In the equation, $\delta {\mathrm{\backslash nolimits}}_{i}^{\min}$ and $\delta {\mathrm{\backslash nolimits}}_{i}^{\max}$ represent the minimum and maximum output coefficients of the unit and represents the output of the unit at each moment in the operation simulation.

2) Power balance constraint

$$\sum_{g=1}^{Ng}P_{g,t}+\sum_{s=1}^{Ns}P_{s,t}+{P\mathrm{\backslash nolimits}}_{r,t}=L_{t}$$ (29)

Where $P_{g,t}$,$P_{s,t}$ and ${P\mathrm{\backslash nolimits}}_{r,t}$ represent the power output of the thermal power unit and the energy storage unit, respectively.

3) ESS operation constraints

$$\left\{ \begin{array}{c} E_{s,t+1}=E_{s,t}+\eta {\mathrm{\backslash nolimits}}_{C}P{\mathrm{\backslash nolimits}}_{C,s,t}\Delta t-\frac{P{\mathrm{\backslash nolimits}}_{D,s,t}\Delta t}{\eta_{D}}\\ E_{s,t=0}=E_{s,t}=T\\ E_{s,\min}\leq E_{s,t}\leq E_{s,\max} \end{array}\right.$$ (30)

$$\left\{ \begin{array}{c} 0\leq P_{C,s,t}\leq \mu_{C,t}P_{C,s,max}\\ 0\leq P_{D,s,t}\leq \mu_{D,t}P_{D,s,max}\\ P_{s,t}=P_{D,s,t}-P_{C,s,t} \end{array}\right.$$ (31)

$$\mu_{C,t}+\mu_{D,t}\leq 1$$ (32)

Where $\eta {\mathrm{\backslash nolimits}}_{C}$ and $\eta_{D}$ are the charging and discharging efficiencies of the ESS; $\mu_{C,t}$ and $\mu_{D,t}$ are binary variables representing the charging and discharging states of the ESS respectively; $P_{C,s,max}$ and $P_{D,s,max}$ are the maximum charging and discharging power,respectively; $E_{s,t=0}$ and $E_{s,t=T}$ are the initial and final energy levels of the ESS, respectively.

## 4. Solution method for bi-level robust planning model

The core idea of this two-stage robust planning model is to optimize for the worst-case uncertainty scenarios (u∈U), ensuring that the planning scheme can satisfy flexibility constraints under all possible uncertain scenarios, especially extreme ones.

The two-stage robust planning model for high-penetration renewable energy systems with increased flexibility can be further transformed into a two-stage three-level optimization problem in the form of min-max-min:

In the first layer (outer minimization), the variable *x* represents the planning decision, which determines the investment capacity of power sources and energy storage systems. In the second layer (maximization), for a given planning decision *x*, the worst-case uncertainty scenario is identified, i.e., the fluctuations in wind-solar output and load *u*. In the third layer (inner minimization), under the given planning decision *x* and uncertainty scenario *u*, the operational variables *y* (such as generator output, charge-discharge status of energy storage, etc.) are optimized to minimize the operational cost.


$$\begin{array}{c} \underset{x}{\min}\left[f_{1}(x)+\underset{u\in U}{\max r(x,u)}\right]\\ \left\{ \begin{array}{c} x={\left[S_{G},S_{W},S_{PV},S_{S}\right]}^{T}\\ y={\left[P_{G},P_{C,S},P_{D,S}\right]}^{T}\\ t=(1,2\cdot \cdot \cdot N_{T}\backslash s.t.\left\{ \begin{array}{c} h(x)\leq 0\\ \left\{ \begin{array}{c} r(x,u)=\underset{y\in \Omega (x,u)}{\min}f_{2}(x,u,y)\\ s.t.\left\{ \begin{array}{c} g(x,u,y)\leq 0\\ l(x,u,y)=0 \end{array}\right. \end{array}\right. \end{array}\right. \end{array}\right. \end{array}$$ (33)

Where $f_{1}$ and $f_{2}$ are the objective functions of the two-stage models, x and y are the decision variables of the two-stage models,u is the uncertain variable containing wind power output, photovoltaic power output, and conventional load, h(x) is the constraint condition of the first-stage investment planning problem, g(x,u,y) is the inequality constraint condition of the second-stage production simulation and operation problem, l(x,u,y) is the equality constraint condition of the second-stage production simulation and operation problem, and Ω(x,u) represents the feasible region of operating variables given decision variables and uncertain variables.

The minimization problem in the second stage is a linear problem in the C&CG algorithm which involves bi-level decision variables. According to the strong duality theory, it can be transformed into its corresponding maximization form and combined with the outer layer maximization problem to obtain the dual problem. The two-stage planning model is then decoupled into a master problem and a sub-problem with a mixed-integer linear form, through this derivation and transformation.

**Fig 2:**
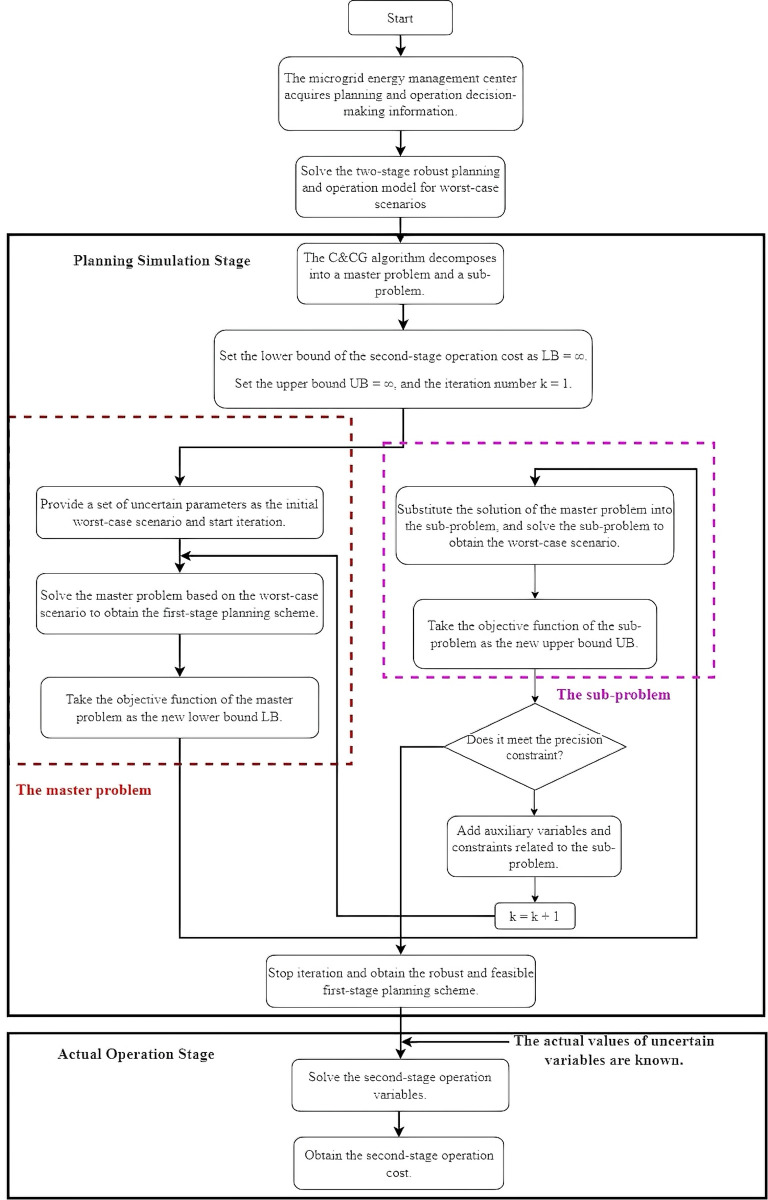
Overview of the C&CG method.

In the master problem, the planning variable *x* determines the investment scheme, while the auxiliary variable *η* represents the estimation of the worst-case operational cost under the currently considered scenario set. In the sub-problem, after fixing x(k), the operational variables *y* are optimized to address the given uncertainty scenario *u*, yielding the worst-case operational cost for the planning scheme. Cutting planes feed back the severe scenario (u(k)) identified in the subproblem and its corresponding operational strategy (y(k)) to the master problem in the form of constraints, thereby transmitting information from the operational level to the planning level. In this way, subsequent solutions to the master problem will avoid selecting planning schemes with excessively high operational costs under the already identified severe scenarios. Through the above iterations, the planning scheme *x* gradually adapts to all potential severe scenarios, thus achieving robustness. Accordingly, the C&CG algorithm can be used afterwards to find a solution. The process is shown in Fig 2:

## 5. Example analysis

### 5.1. Example parameters

The YALMIP toolbox is used to model the problem, and CPLEX 12.9 is called in MATLAB R2021b to solve the model. The power system in a northeastern region of China is selected as a study case. The power system includes photovoltaic, wind power, thermal power, and energy storage. Cost items associated with power exchange with the external power grid are also taking into account from the perspective of long-term planning.

Assuming that no energy storage is installed in the base scenario, Table 2 shows the candidate power sources and energy storage parameters during the planning period. The maximum annual load throughout the planning period is 9000 MW, and the reserve rate is set at 12%. Furthermore, the minimum technical output of thermal power units is 0.6 times their installed capacity. The penalties for curtailed wind and solar power and load shedding are set at 0.1, 0.063, and 20 yuan/kWh, respectively.

**Table 2:** Economic parameters of equipment to be built.

Type	Investment cost (10000 yuan/MW)	Maintenance cost (10000 yuan/MW)	Subsidy cost (10000 yuan/MW)
Thermal power unit	360	20.67	0
PV unit	500	13.71	0
Wind power unit	800	17.69	0
Energy storage unit	120	0.06	0.05

**Fig 3:**
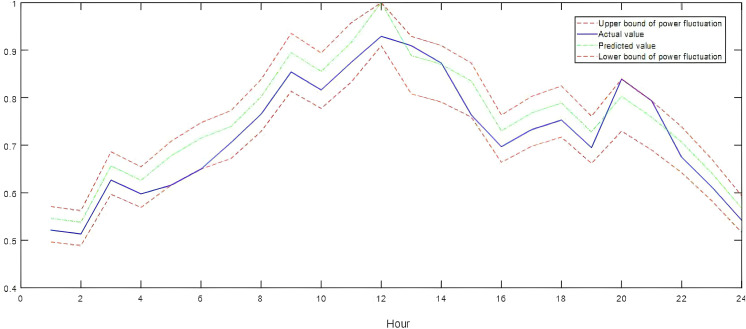
Typical daily load curves.

**Fig 4:**
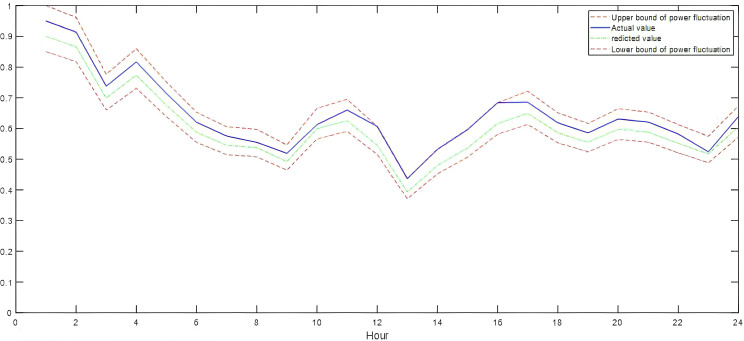
Typical daily wind power production curves.

**Fig 5:**
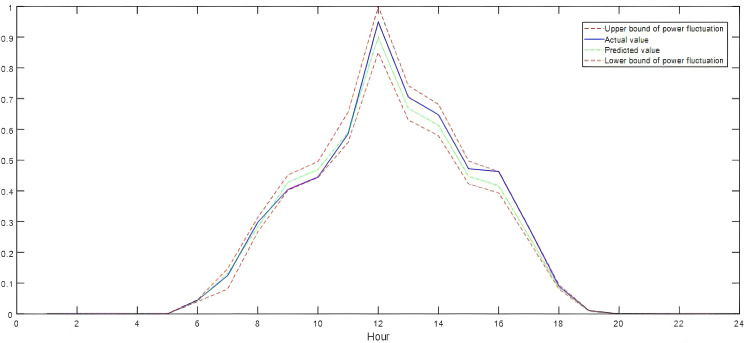
Typical daily solar power production curves.

To address the temporal characteristics of future load and wind/solar power, this study selected ultra-short-term forecast data based on historical temporal characteristics and regional resource endowments. The average prediction errors for wind power, solar power, and load were 15.26%, 14.27%, and 6.89%, respectively. Additionally, the observation-based flexibility demand quantification model was utilized to evaluate the envelope effect of power fluctuations during the dispatch period on a typical summer day, as illustrated in Figs 3–5.

### 5.2. Analysis of the planning results

To confirm the effectiveness of the planning model proposed in this paper, the planning method without the new energy penetration constraint and flexibility constraint was chosen as the benchmark scenario for simulation and comparison. The capacity and planning cost of the system equipment configuration under the current planning model were then calculated.

The planned installation capacity and costs are shown in Table 3. The results preliminarily demonstrate the role of new energy penetration and flexibility constraints. Compared to the conventional planning model, the solution recommended by the flexibility enhancement planning model added 900MW of wind turbines and 100 MW of photovoltaic units, resulting in a reduction of 700 MW of thermal units and a corresponding increase of 14% in overall costs. However, the costs of abandonment and load shedding decreased by 45% compared to Scenario 1, where generation abandonment and load shedding were mandatory means of addressing system inflexibility. The significant reduction in capacity indicates a significant increase in the level of system flexibility.

**Table 3:** Results achieved from using baseline and enhanced planning algorithms.

Planning results	Baseline scenario	Flexibility-enhanced planning
Thermal power unit capacity/MW	4100	3600
Wind unit capacity/MW	2600	3500
Photovoltaic capacity/MW	3900	4000
Cost of electricity curtailment (10000 yuan)	23510	12870
Combined cost(10000 yuan)	608950	671560
Probability of Inadequate Flexibility	7.5%	3.2%

**Fig 6:**
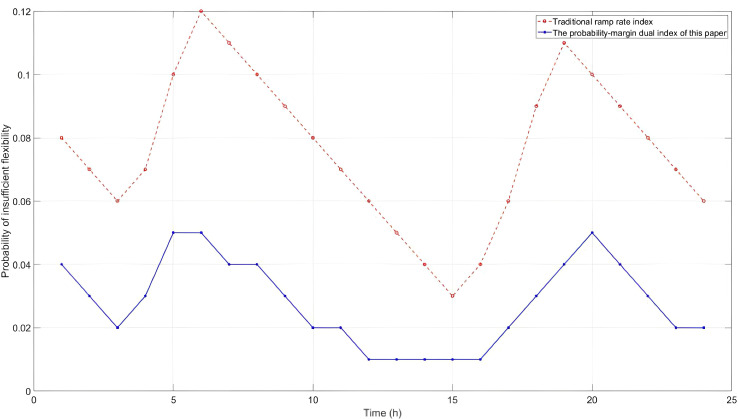
Comparison of system risks under different models.

To verify the effectiveness of the dual-dimensional flexibility index proposed in this paper, Fig 6 compares the probability of insufficient flexibility between the traditional ramp rate constraint model and the model in this paper ver a 24-hour period on a typical day.

Due to neglecting the probability distribution of prediction errors and the joint regulation characteristics among resources, the traditional model exhibits a high probability of insufficiency during peak load periods (08:00–12:00) and periods of sharp decline in wind and solar output (18:00–20:00), with the peak value reaching 12%. In contrast, through dynamic confidence interval adjustment and joint probability integration of multiple resources, the model proposed in this paper reduces the peak probability of insufficiency to 5% and lowers the average daily probability of insufficiency by 57.3%. This fully demonstrates the advantages of the proposed model in the accurate quantification of risks.

### 5.3. Analysis of operating results

In the process of enhancing flexibility in power planning, the operating states of various power units and energy storage equipment in the system are considered after iterative convergence under the constraints of the production simulation process in the two-stage planning model. Additionally, a typical summer day is selected for production simulation analysis of the planning results.

**Fig 7:**
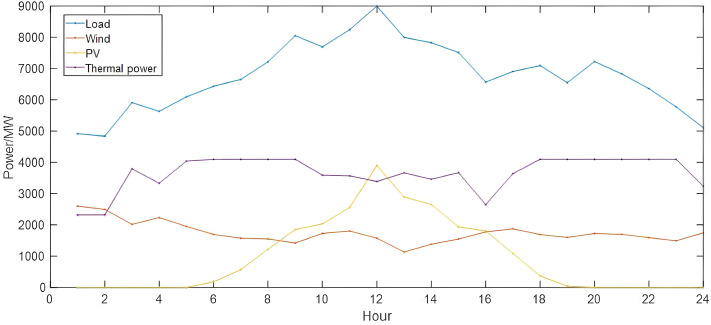
Results of a typical day’s operational scheduling in summer.

Fig 7 illustrates the power output of various types of units in a typical summer scenario. When the wind and solar power output is low, the system relies primarily on thermal power units to maintain balance. Conversely, thermal power units reduce their output when wind and solar power output is high, subject to allowable ramp rates. Renewable energy units play a significant role in the operating strategy, providing most of the power required by the system, while the role of thermal power units shifts towards providing rotational reserves and balancing power.

**Fig 8:**
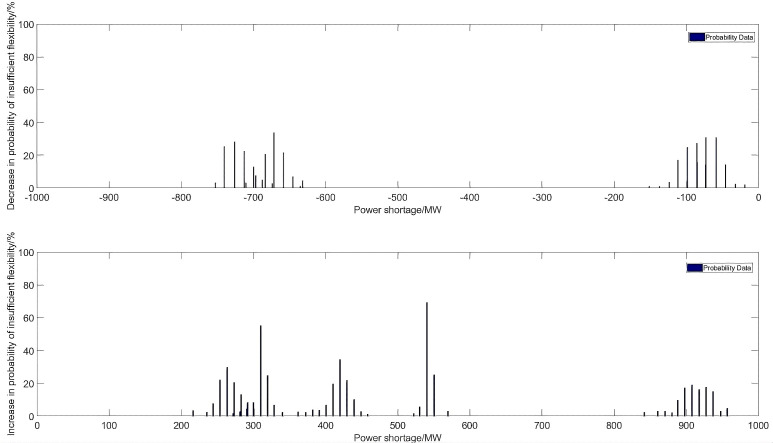
Typical daily probability of inadequate flexibility distribution.

Fig 8 presents the probability of inadequate upward and downward flexibility adjustments. The graph indicates that there is a small probability of large inadequacy in flexibility. However, due to the limitations of thermal units in terms of climbing rate and capacity, they cannot respond instantaneously, and there is a certain probability of inadequate flexibility adjustments both upward and downward. Based on the probability distribution of under-adjustment, it is evident that the overall probability of under-adjustment is smaller than the probability of over-adjustment. This is mainly because there are more instances throughout the day when renewable energy generation is high, resulting in a downward trend in the over-all demand for flexibility. Additionally, since the system must operate economically and cannot respond quickly, the generation of renewable energy during the mid-day period is usually super-imposed on each other, resulting in a greater downward flexibility demand.

To evaluate the impact of the distribution pattern of prediction errors on planning results, this section replaces the normal distribution of wind and solar power prediction errors with an asymmetric Beta distribution with a skewness coefficient of 0.8, while keeping other parameters unchanged. Key indicators such as the probability of insufficient flexibility under the planning scheme are recalculated, and the results are shown in the Table 4.

**Table 4:** Comparison of planning results.

Distribution Type	Baseline scenario	Flexibility-enhanced planning
Normal distribution	7.5%	3.2%
Beta distribution	8.1%	3.5%

As shown in Table 4, when the Beta distribution is adopted: the probability of insufficient flexibility in the benchmark scenario increases from 7.5% to 8.1% (an increase of 8%); the probability of insufficient flexibility in the planning scheme rises from 3.2% to 3.5% (an increase of 9.4%). This indicates that the planning model proposed in this paper is not sensitive to the distribution pattern of errors, and the assumption of normal distribution is applicable within the allowable range of engineering errors.

To explore the impact of confidence interval parameters $\alpha_{r}$ and $\beta_{l}$on planning results, Table 5 presents the comprehensive costs and insufficiency probabilities under different parameter combinations.

**Table 5:** Sensitivity analysis of confidence interval parameters.

$\alpha_{r}/\beta_{l}$	Comprehensive Cost (10000 yuan)	Insufficiency Probability	Risk-Cost Balance
0.80/0.80	658,200	4.1%	Economic priority
0.85/0.85	671,560	3.2%	Optimal balance
0.90/0.90	689,300	2.7%	Safety priority

It can be observed that: when $\alpha_{r}=\beta_{l}=0.80$, the insufficiency probability is relatively high (4.1%), but the comprehensive cost is the lowest; when $\alpha_{r}=\beta_{l}=0.90$, the insufficiency probability decreases to 2.7%, while the comprehensive cost increases by approximately 2.7%; this paper selects $\alpha_{r}=\beta_{l}=0.85$ (corresponding to the Flexibility-enhanced planning), which controls the insufficiency probability at 3.2% with a cost increase of 1.9%, thus achieving the optimal balance between economy and flexibility.

### 5.4. Impact of ESS on planning

To compare the impact of ESS on flexibility enhancement planning, ESS was considered for planning under investment in flexibility resources. Table 6 illustrates the impact of ESS investments on system flexibility enhancement planning. The planning results show that with the inclusion of ESS as a flexibility resource, the output of conventional units will be further reduced, resulting in reduced operating and maintenance costs. The combined cost of Option 3 is reduced by 9% compared to the option without ESS. Furthermore, the abandonment/load shedding cost is further reduced by 27% due to the flexibility regulation capability that the storage system can provide to meet part of the system’s flexibility needs.

**Table 6:** Results of coordinated ESS planning.

Planning results	
ESS capacity/MW	1000
Thermal power unit capacity/MW	3600
Wind unit capacity/MW	2600
Photovoltaic capacity/MW	3000
Cost of electricity curtailment (10000 yuan)	9360
Combined cost(10000 yuan)	650660
Probability of Inadequate Flexibility	2.1%

In high-penetration renewable energy systems, the rapid fluctuations of net load pose severe challenges to the system’s ramping capability. This paper proposes the Ramping Resource Sufficiency Rate indicator to quantify the system’s ability to cope with power changes:


$$\alpha_{t}^{ramp}=\frac{\sum_{i\in G\mathrm{\backslash nolimits}}\Delta P_{i}^{\max}+\sum_{j\in S\mathrm{\backslash nolimits}}r_{j}^{ch/dis}}{\max\left(0,\frac{dL_{t}^{net}}{dt}\right)}$$ (34)

In the equation, G is the set of conventional generators; S is the set of energy storage systems;$\Delta {P_{i}}^{max}$ is the maximum ramping rate of generator i (MW/min); ${r_{j}}^{ch/dis}$ is the charging/discharging rate of energy storage j (MW/min);$\frac{dL^{tnet}}{dt}$ is the net load change rate at time *t* (MW/min). When ${\alpha_{t}}^{ramp}\geq 1$, the system’s ramping capability meets the demand; When ${\alpha_{t}}^{ramp}\geq 1$, there is a risk of ramping deficit.

**Fig 9:**
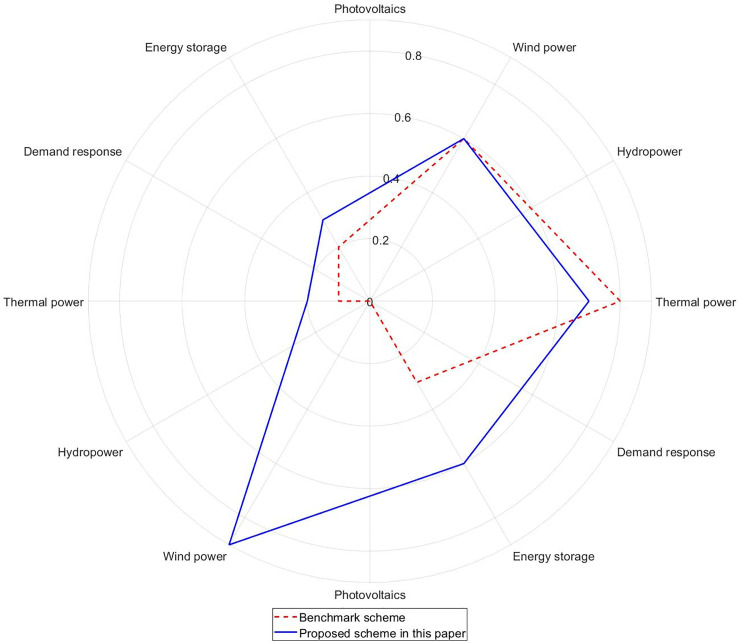
Comparison of ramping contributions of various resources under different schemes.

This paper selects the winter evening peak period (18:00) as a typical scenario, and the ramping contribution of each resource is shown in Fig 9:

Fig 9 shows that in the benchmark scheme, the ramping capability mainly relies on thermal power, but the total sufficiency rate is only 0.8 (<1). The absence of energy storage leads to a ramping deficit when the net load change rate exceeds 100 MW/min. In the improved scheme proposed in this paper, however, the introduced energy storage provides a sufficiency rate contribution of 0.9, accounting for 42% of the total capability. The contribution rate of demand response increases from 30% to 60%, and the total sufficiency rate rises to the critical safety line of 1.0.

### 5.5. Comparative analysis of uncertainty quantification methods

To verify the advantages of the proposed method in uncertainty quantification, a comparison was conducted between the Conditional Value at Risk (CVaR), Information Gap Decision Theory (IGDT), standard Robust Optimization (RO), and the proposed method (robust-probabilistic hybrid model) on the same test system. Specific settings are as follows: for the CVaR method, a confidence level of α = 0.95 was adopted with 1000 scenarios simulated; for the IGDT method, the uncertainty deviation coefficient δ was set to 20%; the standard robust optimization employed a box uncertainty set, with wind and solar output deviations constrained to ±20%. Table 7 presents a comparison of the planning results obtained by different methods.

**Table 7:** Comparison of planning results for different uncertainty quantification methods.

Index	CVaR	IGDT	Standard RO	Proposed Method
Comprehensive Cost (10000 yuan)	693,200	668,500	685,300	671,560
Insufficiency Probability	4.1%	5.2%	3.8%	3.2%
Curtailment Rate	8.7%	12.3%	7.5%	5.9%
Solution Time (min)	52.1	39.8	41.2	38.1

As can be observed from Table 7, the proposed robust-probabilistic hybrid model exhibits significant advantages in uncertainty quantification. Compared with the CVaR method, the proposed method avoids extensive scenario simulation while ensuring risk control through dynamic confidence interval mapping and probabilistic constraints, resulting in a 26.9% improvement in computational efficiency. In comparison with the IGDT method, by quantifying flexibility through dual indices, the proposed method overcomes the conservatism of single extreme scenarios, reducing the curtailment rate by 52.0%. Relative to standard robust optimization, the introduction of probabilistic constraints in the proposed method reduces system risk (insufficiency probability) by 15.8%.

In addition, this paper plots the probability distribution curves of flexibility insufficiency under different methods.

**Fig 10:**
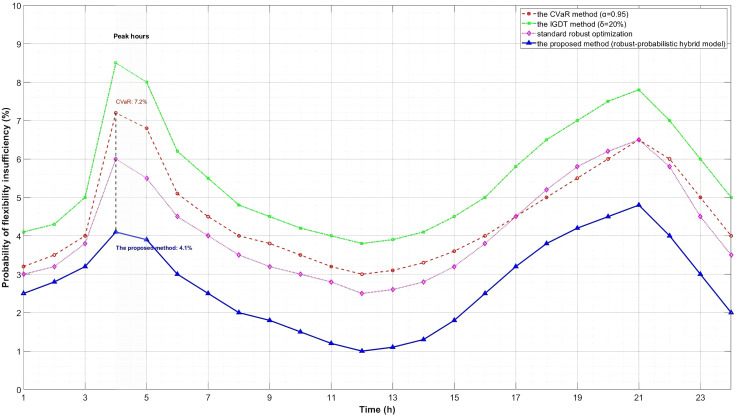
Typical daily probability of inadequate flexibility distribution.

As shown in Fig 10, the insufficiency probability of the proposed method during peak hours (t = 4, 5) is significantly lower than that of other methods, which further verifies the advantages of the proposed method in risk control.

## 6. Conclusions

Based on the analysis of the dynamic balance between flexibility supply and demand in the power system, this paper proposes a dual-index flexibility evaluation metric, specifically flexibility margin and insufficiency probability, to dynamically assess the supply-demand balance under uncertainty. A multi-objective bi-level coordination optimization planning model for flexibility resources is constructed, and the following conclusions are drawn through case applications:

The proposed method effectively coordinates flexible resources including generation, load, and ESS. Implementing flexibility-enhanced planning reduces curtailment/load shedding costs and system flexibility insufficiency probability by 45% and 4.3% respectively. Crucially, incorporating energy storage planning delivers substantial additional benefits: these key metrics are further reduced by 27% and 1.1%. ESS contributes approximately 42% to meeting critical ramping demands, quantified through the Ramping Resource Sufficiency Rate, thereby mitigating challenges from rapid net load fluctuations.

The robust-probabilistic hybrid uncertainty quantification framework demonstrates clear advantages: It achieves 26.9% higher computational efficiency than CVaR by avoiding extensive scenario simulation while maintaining risk control; it reduces curtailment rate by 52.0% compared to IGDT by overcoming conservatism through dual-index quantification; it lowers system risk, specifically insufficiency probability, by 15.8% relative to standard RO through probabilistic constraint incorporation. This framework provides a superior balance between uncertainty handling, risk management, and economy.

## Supporting information

S1 File Minimal dataset flexibility enhanced planning. This Supporting Information file contains the minimal underlying dataset required to reproduce the findings of this study, including: (1) System operation data; (2) Unit equipment parameters; (3) Economic Parameters; (4) Algorithm parameters; (5) Other Parameters. All data are formatted as Excel tables with detailed column annotations for easy use.
(DOCX)

## References

[1] Hand M, Baldwin S, DeMeo E. Renewable electricity futures study. Colorado: National Renewable Energy Laboratory. 2014.

[2] Li J, Ho MS, Xie C, Stern N. China’s flexibility challenge in achieving carbon neutrality by 2060. Renew Sustain Energy Rev. 2022;158:112112. doi: 10.1016/j.rser.2022.112112

[3] AGENCY IE. Harnessing variable renewables. OECD; 2011.

[4] North American Electric Reliability Corporation. Special report: potential reliability impacts of emerging flexible resources[R]. America: North American Electric Reliability Corporation (NERC); 2010. pp. 2–6.

[5] Yasuda Y, Gomez-Lazaro E, Menemenlis N. Flexibility Chart: Evaluation on diversity of flexibility in various areas. In: Proc. of 12th Wind Integration Workshop, October 22-24. London, UK; 2023.pp. 6.

[6] International Energy Agency. Harnessing variable renewables. Paris: International Energy Agency; 2011. pp. 41–67.

[7] Ma J, Silva V, Belhomme R, Kirschen DS, Ochoa LF. Evaluating and Planning Flexibility in Sustainable Power Systems. IEEE Trans Sustain Energy. 2013;4(1):200–9. doi: 10.1109/tste.2012.2212471

[8] Andreas U, Goran A. analyzing operational flexibility of electric power systems. Proc. of Power Systems Computation Conference (PSCC), Aug. 18–22, 2014. Wroclaw. pp. 8.

[9] Zhao J, Zheng T, Litvinov E. A unified framework for defining and measuring flexibility in power system. IEEE Trans Power Syst. 2016;31(1):339–47. doi: 10.1109/tpwrs.2015.2390038

[10] Lannoye E, Flynn D, O’Malley M. The Role of Power System Flexibility in Generation Planning[C]. Power and Energy Society General Meeting. Deteoit, Michgan: IEEE; 2011.

[11] Lannoye E, Flynn D, O’Malley M. Assessment of power system flexibility: A high-level approach[C]. Power & Energy Society General Meeting. IEEE; 2012.

[12] Lu Z, Li H, Qiao Y. Probabilistic Flexibility Evaluation for Power System Planning Considering Its Association With Renewable Power Curtailment. IEEE Trans Power Syst. 2018;33(3):3285–95. doi: 10.1109/tpwrs.2018.2810091

[13] Wen F, Li H, Wen X. Optimal al‐location of energy storage systems considering flexibility deficiency risk in active distribution network. Power Syst Technol. 2019;43(11):3952–62.

[14] Liu Z, Li H, Xuan W, et al. A Multi-scenario Modeling Method for Power System Planning Based on Improved K-Means Aggregation Algorithm[C]. 2022 International Conference on Renewable Energies and Smart Technologies (REST). IEEE; 2022. pp.1–5.

[15] Gao H, Liu J, Wei Z, et al. A bi-level robust planning model of active distribution network and its solution method. Proceedings of the CSEE. 2017;37(5):1389–401.

[16] Roald LA, Pozo D, Papavasiliou A, Molzahn DK, Kazempour J, Conejo A. Power systems optimization under uncertainty: a review of methods and applications. Electric Power Syst Res. 2023;214:108725. doi: 10.1016/j.epsr.2022.108725

[17] Zhao M, Wang Y, Wang X, Chang J, Chen Y, Zhou Y, et al. Flexibility evaluation of wind-PV-hydro multi-energy complementary base considering the compensation ability of cascade hydropower stations. Appl Energy. 2022;315:119024. doi: 10.1016/j.apenergy.2022.119024

[18] Xu L, Huang X, Du Z, Zhang T, Li Z, Cheng L. Research on power planning methods adapting to the development of new energy. Electric Power. 2017;50(09):18–24.

[19] Wang Z, Hou H, Wei R, Li Z. A distributed market-aided restoration approach of multi-energy distribution systems considering comprehensive uncertainties from Typhoon Disaster. IEEE Trans Smart Grid. 2025;16(5):3743–57. doi: 10.1109/tsg.2025.3578484

[20] Wang Z, Hou H, Zhao B, Zhang L, Shi Y, Xie C. Risk-averse stochastic capacity planning and P2P trading collaborative optimization for multi-energy microgrids considering carbon emission limitations: AN asymmetric Nash bargaining approach. Appl Energy. 2024;357:122505. doi: 10.1016/j.apenergy.2023.122505

[21] Lü M, Hu Z. Two stage robust programming of microgrid considering safety margin. Power syst Technol. 2020;44(12):4617–26.

[22] Cheng Y, Zhang N, Lu Z, Kang C. Planning multiple energy systems toward low-carbon society: a decentralized approach. IEEE Trans Smart Grid. 2019;10(5):4859–69. doi: 10.1109/tsg.2018.2870323

[23] Kaushik E, Prakash V, Mahela OP, Khan B, El-Shahat A, Abdelaziz AY. Comprehensive overview of power system flexibility during the scenario of high penetration of renewable energy in utility Grid. Energies. 2022;15(2):516. doi: 10.3390/en15020516

