# Supplementary material for: Research on flexibility-enhanced planning for renewable energy systems with supply-demand uncertainties
Source: PLoS One. 2025 Sep 30;20(9):e0331284. doi: 10.1371/journal.pone.0331284 (PMC12483253; doi:10.1371/journal.pone.0331284)
Supplement: S1 File — This Supporting Information file contains the minimal underlying dataset required to reproduce the findings of this study, including: (1) System operation data; (2) Unit equipment parameters; (3) Economic Parameters; (4) Algorithm parameters; (5) Other Parameters. All data are formatted as Excel tables with detailed column annotations for easy use. (DOCX) [file pone.0331284.s001.docx]

**S1 Minimal Dataset Flexibility Enhanced Planning**

In this paper, the data I used is specifically organized into five tables as follows:

- Table 1: The operating data of the system, including electric load data, photovoltaic power data, and wind power data.
- Table 2: Set equipment parameters of the units in the system.
- Table 3: Economic parameters within the system.
- Table 4: Algorithm parameter data involved in the system.
- Table 5: Other Parameters in the System .

**Table 1 System operation data**

| Time | 1:00 | 2:00 | 3:00 | 4:00 | 5:00 | 6:00 | 7:00 | 8:00 | 9:00 | 10:00 | 11:00 | 12:00 |
| --- | --- | --- | --- | --- | --- | --- | --- | --- | --- | --- | --- | --- |
| Electric  load /MW | 4700 | 4650 | 4600 | 4700 | 5000 | 5400 | 6000 | 6500 | 6900 | 7100 | 7200 | 7200 |
| Photovoltaic power/MW | 0 | 0 | 0 | 0 | 100 | 400 | 900 | 1500 | 2200 | 2700 | 2850 | 2800 |
| Wind power/MW | 3100 | 2900 | 2600 | 2200 | 1900 | 1800 | 1850 | 1950 | 2100 | 2300 | 2450 | 2500 |
| Time | 13:00 | 14:00 | 15:00 | 16:00 | 17:00 | 18:00 | 19:00 | 20:00 | 21:00 | 22:00 | 23:00 | 24:00 |
| Electric  load /MW | 7150 | 7000 | 6800 | 6700 | 6900 | 7500 | 7400 | 7000 | 6200 | 5500 | 5200 | 4800 |
| Photovoltaic power/MW | 2750 | 2500 | 2000 | 1400 | 800 | 300 | 50 | 0 | 0 | 0 | 0 | 0 |
| Wind power/MW | 2550 | 2700 | 3000 | 3200 | 3400 | 3500 | 3400 | 3300 | 3250 | 3200 | 3100 | 3200 |

**Table 2 Unit equipment parameters**

| **Parameters** | **Abbreviation** | **Value** |
| --- | --- | --- |
| Maximum electrical output of thermal power units (MW) | $P_{coal}^{max}$ | 3600 |
| Minimum electrical output of thermal power units (MW) | $P_{coal}^{\min}$ | 2160 |
| Maximum ramping rate of thermal power units (MW/min) | $r_{coal}$ | 72 |
| Maximum electrical output of wind turbine units (MW) | $P_{wind}^{\max}$ | 85 |
| Minimum electrical output of wind turbine units (MW) | $P_{wind}^{\min}$ | 4000 |
| Wind power fluctuation limit (%) | $\Delta P_{wind}$ | ±20 |
| Maximum electrical output of photovoltaic units (MW) | $P_{pv}^{\max}$ | 4500 |
| Minimum electrical output of photovoltaic units(MW) | $P_{pv}^{\min}$ | 0 |
| Photovoltaic fluctuation limit(%) | $\Delta P_{pv}$ | ±20 |

**Table 3 Economic Parameters**

| **Parameters** | **Value** |
| --- | --- |
| Investment Cost of Thermal Power Unit Equipment (10,000 yuan/MW) | 360 |
| Investment Cost of Photovoltaic Unit Equipment (10,000 yuan/MW) | 500 |
| Investment Cost of Wind Turbine Unit Equipment (10,000 yuan/MW) | 800 |
| Investment Cost of Energy Storage System Equipment (10,000 yuan/MW) | 120 |
| Operation and Maintenance Cost of Thermal Power Units (10,000 yuan/MW) | 20.67 |
| Operation and Maintenance Cost of Photovoltaic Power Units (10,000 yuan/MW) | 13.71 |
| Operation and Maintenance Cost of Wind Turbine Units (10,000 yuan/MW) | 17.69 |
| Operation and Maintenance Cost of Energy Storage Systems (10,000 yuan/MW) | 0.06 |
| Abandoned Wind Penalty Cost (yuan/kWh) | 0.1 |
| Abandoned Solar Penalty Cost (yuan/kWh) | 0.063 |
| Load Curtailment Penalty Cost (yuan/kWh) | 20 |

**Table 4 Algorithm parameters**

| **Parameters** | **Value** |
| --- | --- |
| Maximum Number of Iterations for the Column Generation Algorithm | 50 |
| Convergence Tolerance of the Column Generation Algorithm | 0.1% |
| Initial Scenario Set of the Column Generation Algorithm | 10 |
| Time Segment Resolution of the Column Generation Algorithm | 1h |
| Dynamic Confidence Interval of the Column Generation Algorithm | 0.85/0.85 |
| Wind and Solar Power Output Deviation Range in RO Method | ±20% |
| Load Deviation Range in RO Method | ±7% |
| Confidence Level in the CVaR Method | 95% |
| Number of Scenarios in the CVaR Method | 1000 |
| Uncertainty Deviation Coefficient in the IGDT Method | 20% |

**Table 5 Other Parameters**

| **Parameters** | **Value** |
| --- | --- |
| Maximum Annual Load of the System | 9000 |
| Reserve Margin | 12% |
| Network Loss Rate | 10% |
